# Reasons for Individuals not Enrolling for Yoga trial in Addiction

**DOI:** 10.1192/j.eurpsy.2024.729

**Published:** 2024-08-27

**Authors:** S. Sarkar, A. Dhawan, R. Quraishi, P. S. Negi, A. Kumar

**Affiliations:** ^1^National Drug Dependence Treatment Centre, All India Institute of Medical Sciences, New Delhi, India

## Abstract

**Introduction:**

Yoga has been demonstrated to have a range of beneficial effects on individuals with substance use disorders, including opioid use disorders. We initiated a randomized clinical trial to find out the efficacy of add-on yoga among patients with opioid dependence stabilized on treatment to find out whether it led to improvement in sleep and quality of life. However, the rate of enrolment into the study was quite low.

**Objectives:**

In this interim analysis, we present the preliminary data on the reasons for non-enrolment in the yoga trial.

**Methods:**

The single-centre trial involved 1:1 randomization of patients with opioid dependence stabilized on medications (naltrexone or buprenorphine) for a period of at least 4 weeks into two groups (add-on yoga or wait-list control). The yoga included *asanas* and *panchakosha* meditation, taught for a period of 7 days and to be practiced by the participants for a period of 12 weeks. We recorded the reasons for non-participation among those who did not participate and asked them questions about their views on yoga.

**Results:**

Of the 310 patients recruited between August 2022 and July 2023 (99.7% male, mean age 34 years, 56.5% married), 255 (82.3%) could not be enrolled in the trial. The most common reasons for non-enrolment were not having time for training (n = 206, 80.8%), not having time for doing yoga (n = 180, 70.6%), not having a smartphone for continued training or contact (n = 31, 12.2%), distance from the center (n = 17, 5.5%) do not feel the need for yoga (n = 16, 5.2%), injury or disability (n = 9, 3.5%), old age or medical condition (n = 7, 2.7%), already doing gym exercises (n = 7, 2.7%), nature of job (n = 5, 2.0%), do not have knowledge of yoga (n = 5, 2.0%), and do not think yoga would be useful (n = 4, 1.6%). Among those who could not be enrolled, 35.1% reported doing yoga sometime in the past, and 21.6% reported that at least one of the family members did yoga. When asked whether they would be interested if yoga was available online, 16 (5.2%) responded ‘yes’ and 45 (14.5%) responded ‘maybe’.

**Conclusions:**

Expressed time constraints may be an important factor deterring patients with opioid dependence from engaging in yoga as an add-on yoga. There are other reasons as well that may deter patients from such an intervention. The findings should be seen in the light of the limitation of a single medically oriented center, and patients already stabilized on treatment.

**Disclosure of Interest:**

None Declared

